# TOMAS-R: A template to identify and plan analysis for clinically important variation and multiplicity in diagnostic test accuracy systematic reviews

**DOI:** 10.1186/s41512-022-00131-z

**Published:** 2022-09-22

**Authors:** Sue Mallett, Jacqueline Dinnes, Yemisi Takwoingi, Lavinia Ferrante de Ruffano

**Affiliations:** 1 UCL Centre for Medical Imaging, University College London, London, W1W 7TY UK; 2 Test Evaluation Research Group, Institute of Applied Health Research, University of Birmingham, Edgbaston, Birmingham, B15 2TT UK; 3 NIHR Birmingham Biomedical Research Centre, University Hospitals Birmingham NHS Foundation Trust and University of Birmingham, Birmingham, UK; 4 York Health Economics Consortium, Enterprise House, Innovation Way, University of York, York, YO10 5NQ UK

**Keywords:** Diagnostic test accuracy, Systematic review, Multiplicity, Heterogeneity, Meta-analysis, forest, SROC, Methodology, Template

## Abstract

The Cochrane Handbook for Systematic Reviews of Diagnostic Test Accuracy (DTA) provides guidance on important aspects of conducting a test accuracy systematic review. In this paper we present TOMAS-R (Template of Multiplicity and Analysis in Systematic Reviews), a structured template to use in conjunction with current Cochrane DTA guidance, to help identify complexities in the review question and to assist planning of data extraction and analysis when clinically important variation and multiplicity is present. Examples of clinically important variation and multiplicity could include differences in participants, index tests and test methods, target conditions and reference standards used to define them, study design and methodological quality. Our TOMAS-R template goes beyond the broad topic headings in current guidance that are sources of potential variation and multiplicity, by providing prompts for common sources of heterogeneity encountered from our experience of authoring over 100 reviews. We provide examples from two reviews to assist users. The TOMAS-R template adds value by supplementing available guidance for DTA reviews by providing a tool to facilitate discussions between methodologists, clinicians, statisticians and patient/public team members to identify the full breadth of review question complexities early in the process. The use of a structured set of prompting questions at the important stage of writing the protocol ensures clinical relevance as a main focus of the review, while allowing identification of key clinical components for data extraction and later analysis thereby facilitating a more efficient review process.

**Supplementary Information:**

The online version contains supplementary material available at 10.1186/s41512-022-00131-z.

## Background

Systematic reviews are widely recognised as the best way of summarising current evidence on a particular research question [1]. To be clinically relevant, systematic reviews need to have a clear research question and pre-specified review methods based on a detailed understanding of both clinical pathway and clinically important issues within the review question [2, 3]. Diagnostic test accuracy (DTA) systematic reviews can have additional complexities compared to intervention systematic reviews. These arise from all parts of the review but frequently occur due to the inclusion of multiple index tests, reference standards and sometimes multiple test thresholds [4]. In addition the variation in participants and their disease state may be greater than is found in intervention reviews, as DTA reviews can include a range of disease severity and participant groups.

Most DTA systematic reviews have clinical and statistical complexities that require careful and robust planning to allow pre-specification of analysis and to avoid additional data extraction at a late stage in the review because data and analysis complexities were not identified during protocol development. In particular, taking proper account of complexity in the data structure is important for appropriate statistical analysis [5]. The Cochrane Screening and Diagnostic Tests Methods Group provides a range of resources to assist in the preparation of a DTA systematic review [6]. The Cochrane Handbook for Systematic Reviews of Diagnostic Test Accuracy [4], supported by Cochrane DTA online learning modules (https://methods.cochrane.org/sdt/dta-author-training-online-learning), includes help on how to develop a systematic review question, in terms of population, target condition, index test(s) and reference standard(s) within the context of a clinical pathway. The Cochrane Review Manager software is a review authoring tool that includes a template for writing a DTA systematic review protocol, with prompts for the key areas which need defining in a review.

The TOMAS-R template (Template of Multiplicity and Analysis in Systematic Reviews) is intended to ensure the clinical relevance of a systematic review and to enable a more efficient review process. TOMAS-R goes beyond the broad topic headings provided in current guidance, providing a structured format with prompting questions, to help identify complexities in the review question and to assist planning of data extraction and analysis when clinically important variation and multiplicity is present. A thorough understanding of any inherent complexities and a clear plan for dealing with them are important to maintain the clinical relevance of the review and to understand heterogeneity in the evidence base. The template is intended to be used at the important stage of writing the protocol, with the aim of increasing reliability and efficiency at later stages of the review process. In our experience, failure to identify the full breadth of review question complexities early in the process causes considerable additional work, compromising efficiency.

We provide this template and guidance with examples from two DTA systematic reviews with an aim to enhance the quality and consistency of DTA protocols and reviews. The TOMAS-R template is intended to be used alongside existing guidance for DTA reviews provided in the Cochrane Handbook.

## Objectives

Our objective is to provide a template to help review authors identify the critical sources of clinically important variation and multiplicity in a DTA review question, and to consider the implications for data extraction and analysis. This template aims to facilitate communication between methodological, clinical and patient/public review team members, to ensure important clinical complexities are identified at the start of a review, ideally during protocol development.

## Methods

We developed this template based on our experience as authors and reviewers of more than 100 DTA reviews. The tool was piloted by colleagues with expertise in methodology, statistics and systematic reviews, both individually and in seminars (e.g. Test Evaluation Research Group seminar at University of Birmingham 2016) and workshops (Systematic reviews and meta-analyses of diagnostic test accuracy in 2017, 2018, 2019), and using examples of reviews in different clinical areas. The template was piloted and adapted using two reviews (SM) and was also employed as a peer review tool for a series of Cochrane DTA reviews (SM 2017 to 2019). We elicited feedback as edits on the template, verbally and in email feedback on our proposed uses for the template, and ways to improve its usefulness. The author group refined the template and elaboration during article preparation and additional suggestions raised by article reviewers during the peer review were incorporated.

Feedback on the first draft related to (a) content: addition of variation by risk of bias (QUADAS2); addition of prior symptoms and prior treatment as part of participant characteristics; adaption to allow multiple diseases (b) presentation: headings; ordering of sections (c) explanation to new users: presentation of example templates; explanations; modification of examples; improvements to wording (d) intended use: consideration of patient involvement; consideration of how the template might be used for different types of review (intervention, prognostic, exploratory, scoping); how the template could be used in peer review; to record differences between the protocol and the final review.

### TOMAS-R template

The TOMAS-R template is based on the recognition that although every review is different, there are common issues that often underlie important clinical differences and variations affecting the clinical applicability of a systematic review. We recommend TOMAS-R should be used for protocol development during and after initial discussions with clinical colleagues to identify the complexities of the review question, objectives and study eligibility criteria, and after some scoping searches of the literature have been performed. We recommend that one or two example primary studies likely to be included in the review are used alongside TOMAS-R to generate and guide discussion.

The tool sets out five steps to be followed across four key domains for any DTA review: participants, index test(s), target condition and study design. We illustrate each domain using worked examples from two DTA systematic reviews, one on rapid tests for diagnosis of typhoid [7], and one on biomarker tests in ovarian cancer [8], supplemented with reference to other reviews to illustrate specific points. The five steps are set out in Table 1, and Table 2 presents a full template example for the review of rapid diagnostic tests for detection of typhoid. A blank template table is provided in supplementary materials (Table S1).

**Table 1:** Summary of TOMAS-R steps

Step 1	Summary of review objectives and proposed eligibility criteria Set out key review objectives with broad definition of study participants, target condition and reference standard, index test(s) and study design
Step 2	Scoping potential complexities resulting from clinically important variation and multiplicity. Identify and record potential complexities that could ultimately affect how data are extracted, presented and combined. Examples of variation could include differences in participants, index tests and their methods, target conditions and reference standards used to define them, study design and methodological quality.
Step 3	Simplify the review whilst maintaining clinical relevance For each potential source of variation (complexity) consider whether differences in test accuracy might be observed. Consider whether separate analysis or heterogeneity investigation is appropriate
Step 4	Planning data extraction Develop and pilot a standardised data extraction sheet. Define any data or categories of data to be preferentially extracted, e.g. by participant group, by definition of target condition, by index test method or threshold
Step 5	Planning presentation and analysis of data Record plan for meta-analysis. Record how data complexity will be presented using graphs, tables, and additional analyses such as investigation of heterogeneity or sensitivity analysis where appropriate and feasible with available data. Recommended graphical presentation includes summary ROC (SROC) plots with individual study data and summary estimates of sensitivity and specificity (summary point) with 95% confidence and prediction regions.

**Table 2:** Example TOMAS-R template to identify clinically important variation and multiplicity for typhoid review

**Summary of review (step 1)** **Title:** Rapid diagnostic tests for typhoid and paratyphoid (enteric) fever **Primary objective:** to assess the diagnostic accuracy of rapid diagnostic tests (RDTs) for detecting enteric fever in persons living in endemic areas presenting to a healthcare facility with fever. **Secondary objectives:** To identify which types and brands of commercial test best detect enteric fever To investigate the sources of heterogeneity between study results including: *Salmonella enterica* serovars (Typhi or Paratyphi A); case-control study design; test population; reference standard; index test format; index test sample; population disease endemicity; participant age; geographical location.				
**Participants:** Clinically-suspected enteric fever patients or unselected febrile patients **Index test(s):** All rapid diagnostic tests specifically designed to detect enteric fever cases; applied to patient blood or urine samples **Role of test for patient** (delete as appropriate diagnosis, monitoring, staging): diagnosis **Role of test in planned clinical pathway:** (delete as appropriate triage, add on, replacement): replacement **Target condition:** typhoid and paratyphoid (enteric) fever **Reference standards:** bone marrow culture, peripheral blood culture, peripheral blood culture, and polymerase chain reaction (PCR) on blood **Study designs:** prospective cohort, retrospective case control				
**Domain 1: Participants**				
**Potential sources of clinically important complexity**	**STEP 2: List categories identified from review scoping**	**STEP 3: Report which categories will be separate or combined. Give Reason**	**STEP 4: Data extraction. Report if any categories will be preferentially extracted.**	**STEP 5: Presentation and meta-analysis. Report how categories will be treated**
**1.1: Clinical pathway/prior tests/different comorbidities/geographical regions** Are there important differences between participants that could affect test accuracy? Examples • Different clinical pathways or healthcare settings (primary care, secondary, tertiary care) • Different prior tests (referral based on different prior tests) • Differences in other conditions likely to be present at same time • Different geographical settings	**Clinical pathway/prior tests** Two groupings: • clinically-suspected enteric fever • unselected febrile patients • some studies may include a mixture of patients	Keep as separate groups if possible. Retain studies with mixed or unclear populations. Report grouping based on study inclusion criteria in TOC. Reason: studies could include populations with varying pre-test probabilities of disease, or other concomitantly circulating infectious diseases	Preferential data extraction in separate groups^b^. Otherwise extract as a mixed population group.	Planned SROC or forest plots with groups indicated for each study. Planned heterogeneity analysis.
	**Level of disease endemicity** Use two groups for level of disease endemicity to take account of pre-test probability of disease (e.g. medium versus high using classification of Crump 2004).	Keep as separate groups if possible. Report grouping based on study inclusion criteria in table of study characteristics. Use prevalence in study as measure of endemicity if not otherwise reported. Reason: tests have potential for varying performance in endemic and non-endemic regions	If a study includes data from two different endemicity disease levels separately (e.g. different centres or different seasons) preferentially extract data in separate groups. Where not reported, or populations are mixed, use study prevalence as proxy for endemicity.	Planned SROC or forest plots with groups indicated for each study or ordered by individual study prevalence. Planned heterogeneity analysis.
	**Geographical location** Use two groups (sub-Saharan Africa versus the rest of the world).	If sufficient studies then keep separate, otherwise combine. Report country in TOC Reason: in sub-Saharan Africa non-typhoidal Salmonellae are an important cause of bacteraemia; may affect the performance of enteric fever RDTs in this region.	Preferential data extraction in separate groups. Otherwise extract as a mixed population group.	Planned SROC or forest plots with groups indicated for each study. Planned heterogeneity analysis.
**1.2: Disease type or severity** Are there groupings within participants by disease type or severity that could affect test accuracy? Examples • Different severity of disease: patients with mild disease vs with severe disease • Different disease state, e.g. active vs past disease (inactive) • Different types of diseased, e.g. pigmented vs non-pigmented lesions in skin cancer	Not considered in this review	Not applicable in this review Reason: diagnosis is presence of typhoid disease. Severity of disease is measured by patient symptoms and signs.	Not applicable in this review	Not applicable in this review
**1.3: Participant demographics** Are there any important groupings by participant age, gender, ethnicity? Example separate groups by • Different ages such as children and adults • Different demographics such as gender, ethnicity, genetic groups	Two groups by age • Adult (over 16 years) • children (16 years or younger)	If sufficient studies then keep separate, otherwise combine. Reason: test might perform differently in children and adults, in part due to different prevalence of other infectious diseases.	Preferential data extraction in separate groups. Otherwise extract as a mixed population group.	Planned SROC or forest plots with groups indicated for each study.
**Domain 2: Index test(s)** **Criteria used to focus review to most clinically relevant test(s)**:				
**Reason for potential groupings or categories.**	**STEP 2: List categories identified from review scoping**	**STEP 3: Report which categories will be separate or combined. Give reason**	**STEP 4: Data extraction. Report if any categories will be preferentially extracted.**	**STEP 5: Presentation and meta-analysis. Report how categories will be treated**
**2.1 Type of underlying index test** Is more than one underlying type of index test included that could affect test accuracy? Examples • Different indications of disease presence e.g. DNA of infectious agent, antibodies against infectious agent. • Different formats of test, e.g. ELISA, PCR, dipstick • Different equipment needed that affect test e.g. laboratory test using specialist equipment, point of care test.	3 main commercial tests 1. Typhidot 2. TUBEX 3. KIT Other tests include PanBio Multi-test Dip-S-Tick, Mega Salmonella and SD Bioline tests	Separate groups for main tests. Variations within a test are grouped together 1. Typhidot 2. TUBEX 3. KIT All other tests considered separately include PanBio Multi-test Dip-S-Tick, Mega Salmonella and SD Bioline tests Reason: Identified 3 tests where sufficient studies to consider meta-analysis. Meta-analysis across other tests using different tests and test approaches not useful for review	Extract all test data from each study.	Separate meta-analysis for each commercial test^a,c^. Where insufficient number of studies for meta-analysis, then graphical data presentation with descriptive analysis. Where sufficient number of studies available, make comparison between tests.
**2.2 Index test methods within an index test grouping** Is there more than one method or manufacturer for a test that could affect test accuracy? Also consider if the test might be done by people with different level of experience or using different approaches to interpretation. Examples • Different test versions of tests • Different participant samples used to detect disease, e.g. blood sample, urine sample • Differences in staff, e.g. trained laboratory staff vs nurse point of care test • Different treatment of inconclusive test results • Different approach to assist test interpretation, e.g. algorithms or checklists	**Different test versions** 1. Typhidot; Typhidot-M; TyphiRapid Tr-02 - grouping within Typhidot of IgM or IgG Ab detection 2. KIT: dipstick assay; latex agglutination assay; lateral flow immuno-chromatographic test 3. TUBEX: 1 format	Different versions within a test will be combined Reason: main review question about accuracy of 3 main test types.	Record test version in TOC.	All test versions combined in meta-analysis as single group^a,b^.
	**Different samples**	Separate by sample type Reason: sample type considered important for test results	Separate data extraction by sample type	Planned heterogeneity analysis if sufficient studies. In review, no heterogeneity analysis as all studies use blood samples
	**Different treatment of inconclusive results**: Typhidot test only	Will combine Typhidot tests regardless of treatment of inconclusive results. Reason: Most studies report results for Typhidot such that IgM results can be extracted, so expect data extraction to standardise inconclusive results reporting.	For Typhidot, we extracted IgM in preference to IgG. Reason: IgM indicates recent infections whereas IgG can pick up previously resolved infections. Using Typhidot IgM allowed better comparison with TUBEX and KIT as these tests both detect IgM antibodies	Individual results will be presented in SROC plot labelled by method of treatment of inconclusive results. Main analysis across all Typhidot^a,c^, but with sensitivity analysis limited to those reporting inconclusive results or where test format means there are no inconclusive results. Reason: different treatment of inconclusive results could influence results
**2.3 Threshold(s) for positive index test result** Are different thresholds used to define a positive result that could affect test accuracy? Has a clinically relevant index test threshold been identified for this review? Examples • Different test thresholds used to define a positive test result for semi-quantitative or continuous test results	Some KIT tests provide semi-quantitative test results where different thresholds can be used to define positive test results. Other test formats provide qualitative test results without any thresholds.	Keep KIT thresholds separate. Main result for KIT based on threshold of > 1+ which was judged the most meaningful clinically. Reason: reporting results at clinically relevant test threshold(s) is most important result for clinical practice. Results combined across very different thresholds do not give a result that can be interpreted at any clinically relevant threshold, but correspond to an average result reflecting how often different thresholds are reported.	Results extracted separately for each KIT test threshold. KIT threshold of > 1+ was judged the most meaningful clinically	Meta-analysis undertaken for the threshold of > 1+ only, as this was judged the most meaningful^a,c^. Individual study results presented in SROC graphs
**Domain 3: Target condition**				
**Reason for potential groupings or categories.**	**STEP 2: List categories identified from review scoping**	**STEP 3: Report which categories will be separate or combined. Give reason**	**STEP 4: Data extraction. Report if any categories will be preferentially extracted.**	**STEP 5: Presentation and meta-analysis. Report how categories will be treated**
**3.1 Types of target condition** Are there different target conditions included that could affect test accuracy? Examples • Different causes of disease (e.g. different organisms causing typhoid infection, different causes of trauma injury) • Different types or severity of disease that are treated differently, e.g. malignant and borderline disease in ovarian cancer disease diagnosis, any melanoma or melanoma with high potential to progress to malignancy	Salmonella typhi or Paratyphi A	Keep as separate groups if possible. Retain studies with mixed or unclear populations. Reason: tests likely to perform differently for different bacteria and bacterial subtypes	Preferential data extraction in separate groups. Otherwise extract as a mixed population group.	Protocol planned heterogeneity analysis if sufficient number of studies. *In review, no heterogeneity analysis as all studies of Salmonella typhi infection*
**3.2 Reference standards** Are different methods used to verify disease presence or absence that could affect test accuracy? Examples • Different methods to detect typhoid infection measured by detection of viral DNA or by bacterial culture	Four methods: bone marrow culture, blood culture, PCR peripheral blood, combinations of tests. For labelling of studies, two grouping of reference standards are defined. Grade 1 study was defined as one using both bone marrow culture and peripheral blood culture. A Grade 2 study was defined as using either peripheral blood culture only, or peripheral blood culture and peripheral blood PCR as the composite reference standard.	Keep reference standards separate. Categorise as grade 1 or grade 2 for TOC. For subsequent analysis it may be important to compare different reference standards within studies where possible. Reason: reference standards are likely to have differing ability to detect low levels of infection (blood vs bone marrow) and also to be able to differently detect live untreated (both culture and PCR), treated (unlikely with culture but should detect with PCR) and dead bacteria (not culture but should detect with DNA).	Extract all test data from each study, so index test data maybe extracted for two or more reference standards.	Meta-analysis using most commonly used reference standards as priority^a,c^. Planned SROC or forest plot with groups indicated for each study. Planned heterogeneity analysis if sufficient number of studies. *In review, no heterogeneity analysis as insufficient studies, but SROC plot displayed data for different reference standards.*
**3.3 Thresholds for reference standard** Are different criteria or thresholds used to define presence of disease that could affect test accuracy? Examples • Different definitions of fasting blood glucose to define diabetes	Not applicable in this review	Not applicable in this review Reason: infection classified as present or not	Not applicable in this review	Not applicable in this review
**3.4 Time of reference standard determination** Are there differences in when reference standard is completed that that could affect test accuracy? Examples • Different time points reference standard assessed • Different maximum or minimum time intervals between reference standard and index test	Not applicable in this review	Not applicable in this review Reason: index test and reference standard are determined at a single time point.	Not applicable in this review	Not applicable in this review
**Domain 4: Study design and quality**				
**Reason for potential groupings or categories.**	**STEP 2: List categories identified from review scoping**	**STEP 3: Report which categories will be separate or combined. Give reason**	**STEP 4: Data extraction. report if any categories will be preferentially extracted.**	**STEP 5: Presentation and meta-analysis. report how categories will be treated**
**4.1: Unit of analysis:** Are there differences in whether test results are a single test or more than one test result per participant (unit of analysis)? Examples • Test results could refer to individual participants, lesions, organ, clinic visits or imaging scans	Per participant	Not applicable Reason: In this review all results were reported based on disease status of patients	Not applicable	Not applicable
**4.2: Risk of bias: QUADAS-2/QUADAS-C item or domain** Based on risk of bias assessed using QUADAS-2/QUADAS-C, are there important differences between studies that could affect test accuracy? Examples • Single signalling question, e.g. specific design criteria (case control vs better design using cohort or nested case control) • Differences in QUADAS-2/QUADAS-C overall domain assessment of bias, e.g. participant domain	Study design: case control, prospective cohort, randomised controlled trial, paired comparative trial	Decision to retain all study designs in main analyses and to graphically present variation in study design. Reason: In this review, test type and test threshold prioritised as sources of variation, due to the limited number of studies. The bias due to case-control design will be presented graphically. Case-control studies were only included where controls consisted of patients with similar clinical presentation. Case-control studies with most extreme bias, due to use of healthy control patients, were excluded from the review.	Only one group of data at study level.	Planned SROC plot with groups indicated for each study. Protocol planned heterogeneity analysis if sufficient number of studies. In review, no heterogeneity analysis but descriptive comment.
**4.3: Applicability: QUADAS-2 item or domain** Based on applicability of study results assessed using QUADAS-2, are there important differences between studies that could affect test accuracy? Applicability of participants could depend on several factors and might be best summarised by analysis grouped by applicability of the participant recruitment assessed in QUADAS-2 **Example** Differences in QUADAS-2 domains for applicability of: • Participants • Index tests • Reference standard	Not considered	Not applicable Reason: main biases from individual QUADAS-2 domains are addressed in presentation and analyses above. Participant domain: case control vs cohort presentation Index test: threshold bias addressed. Interpretation of inconclusive results addressed. Reference standard domain: 3 grades of reference standard addressed. Flow and timing: Verification bias not applicable, time intervals not issue in review, inconclusive results included missing data issues	Not applicable	Not applicable

The first section is where the summary of the review is reported (step 1 of TOMAS-R), including the review title, objectives and components of PICO adapted for a diagnostic accuracy review. Steps 2, 3, 4 and 5 are reported in separate columns of the TOMAS-R template for each domain (participants, index test, target condition and study design and quality. Within each domain the first table column describes and gives examples of potential sources of clinically important complexity commonly found in reviews. This allows a systematic discussion of potential sources and reporting of identified complexities alongside data extraction and analysis decisions and rationale used to handle these complexities. The template is filled in for the rapid typhoid review. A blank template is included in the supplementary appendix

*Abbreviations*: *DNA* deoxyribonucleic acid, *ELISA* enzyme-linked immunosorbent assay, *PCR* polymerase chain reaction, *QUADAS-2* quality assessment of diagnostic accuracy studies, *RDTs* rapid diagnostic tests, *ROC* receiver operating characteristic, *SROC* summary receiver operating characteristic, *TOC* table of study characteristics

^a^Meta-analysis will only be done if (i) there are four or more studies where results are given in the same format (e.g. 2 × 2 table for diagnosis) (ii) study results are sufficiently homogeneous visualised in forest plots or ROC space for a meaningful representation by a single summary statistic.

^b^Priority order of data extraction means that not all data will be extracted from published articles.

^c^To avoid over representing results from a study in meta-analysis results, we will include only one set of results per index test from each study

## Step 1: Summary and review objectives

In step 1 of TOMAS-R, a summary section lists the main review question headings, which allows the title, primary and secondary objectives of a review to be recorded, including a broad outline of participants, index test(s), target condition and study design.

## Step 2: Scoping potential complexities

At step 2 each of the four domains is considered in turn, in order to identify and record sources of complexity. A number of subsections representing key sources of possible variation and multiplicity are suggested for each domain, each featuring a prompt to discuss whether it applies to the current review question. 

This template could also be used to identify and record how the scoping of the review is affected by the purpose of a review and the funder. For example, reviews commissioned by the National Institute for Health and Care Excellence, the World Health Organisation, the National Institute for Health Research (NIHR) or published by Cochrane, may have a different focus.

### Domain 1: Participants

For participants in a study, the template in Table 2 highlights three important components for review scoping (1) the clinical pathway and setting, including prior tests, comorbidities and geographical region (2) the severity of disease and (3) participant demographics.

The point on the clinical pathway at which a test is used in patient management affects the composition of the participant group receiving the test, largely because the quantity and type of tests a person receives before the index test modifies the likelihood of having the target condition. For example, tests to detect typhoid can be used both in people with an a priori clinical suspicion of enteric fever and in those with fever but without any clear suspicion of typhoid. Similarly, geographical location and the level of disease endemicity of participants were identified as potentially important to understand the applicability of study results. Geographical region and the level of endemicity influences the background level of typhoid amongst competing infectious agents potentially causing fever and can also distinguish the type of bacterial infection underlying typhoid.

In the review of tests for ovarian cancer, scoping suggested that the CA125 test was likely to perform differently in pre- and post-menopausal women. As menopausal status can be established in a simple patient history or approximated by age, it was important to provide separate estimates of accuracy by menopausal status. This required separate data extraction of results by menopausal status, and in this review, exclusion of studies where separate results were not available. Analysing results separately according to disease severity corresponding to cancer stage was also determined likely to be clinically relevant; however, few studies identified during scoping provided separate results by stage, so separate data extraction and analysis was not attempted.

### Domain 2: Index test(s)

The potential for variation in the index test is common. While a review question is usually focussed on the accuracy of a generic diagnostic test type (for example ‘rapid diagnostic tests’ for detecting enteric fever), in reality many different tests may exist within a generic class of tests for a specific purpose. How we define what constitutes a similar enough test to allow clinically meaningful grouping, and which variations in the test should be analysed separately, are integral to producing aggregate estimates of test accuracy that answer the systematic review objective and are clinically useful, generalisable and methodologically valid.

TOMAS-R highlights and provides prompts (Table 2) for three common potential causes of variation and complexity in the review index test(s): (1) different types of index tests; (2) different methods (including differences in test versions, manufacturers, sampling methods, staff training, treatment of inconclusive test results or methods used to assist test interpretation); and (3) different thresholds to define a positive index test result.

In the typhoid review, scoping identified several different rapid tests in use including three main commercial tests. Test methods were different between studies including variations in: manufacturer test versions; samples used; and index test thresholds for one test. For the purpose of the review, it was considered important to summarise each commercial test separately because the assay formats were different (ELISA, lateral flow, magnetic bead), and differences in the type of antibody detected meant that tests would have different time spans of detection post infection (IgM or both IgM and IgG); however, variations within the same commercial brand of test were grouped together. For the KIT test, the most clinically relevant threshold was identified as greater than 1. In the protocol, it was recognised that rapid tests could use either blood or urine samples; data extraction planned to record the type of test sample to allow separate presentation, if sufficient results were available.

In the ovarian cancer review, tests were grouped by the biomarker type, for example HE4 biomarker, with different commercial tests analysed together in the same group as the review focussed on identifying which biomarkers were potentially useful, rather than which specific test brand was the most accurate. Tests used different biomarker thresholds to define a positive test result, so the review focussed on a small number of pre-specified commonly used test thresholds for each biomarker. Data extraction was limited to results using thresholds within a small range of values around the pre-specified test thresholds. At these pre-specified test thresholds, average sensitivity and specificity were estimated using meta-analysis methods based on a single result per study. In future reviews, if newer meta-analysis methods that allow multiple thresholds from each study to be combined in a single analysis are planned [9, 10], then all thresholds would need to be extracted.

### Domain 3: Target condition

Differences in how the presence of the target condition (or disease) is defined can vary between studies, affecting measurement of diagnostic accuracy. The potential for variation and complexity in the review target condition is influenced by four components: (1) different types of target condition, (2) different reference standards, (3) different severities of the target condition (reference standard thresholds) and (4) differences in the time interval between the index test and reference standard.

In the systematic review of typhoid tests, there are two different organisms that can cause enteric fever, typhoid caused by Salmonella typhi and paratyphoid caused by Paratyphi A. Ideally test accuracy would be examined separately for each type of typhoid, however this was not expected to be possible due to small numbers of studies examining these forms of typhoid separately. From scoping, three main reference standards were identified and preferentially ranked for analysis: bacterial culture using samples from (1) bone marrow culture; (2) blood culture; or (3) blood sample PCR assays which in some studies were interpreted in combination with bacterial culture from blood samples. If a study reported data for an index test against more than one reference standard, all data were extracted. This enabled comparisons between index tests to be restricted to studies using the same reference standard.

In the ovarian cancer review, two target conditions are recognised as malignant and borderline disease. The focus of the review question was to identify women as having disease defined as either malignant or borderline, compared to no disease defined as benign. Scoping identified that primary studies considered borderline disease in different ways; some studies grouped borderline with malignant disease, others grouped borderline with benign and some studies excluded women with borderline disease. Consequently, separate data extraction for different definitions of target condition was planned, allowing a focus on studies that were most applicable to the review, and investigation of how study results were affected by different reference standard choices.

The ovarian cancer review included studies with different time intervals between the index test and reference standard, affecting results for women where clinical follow-up was the reference standard. Clinical follow up is the reference standard for women who do not attend surgery for ovarian disease, and therefore do not have a histology reference standard. Differences in clinical follow up needs consideration as this can affect test accuracy.

### Domain 4: Study design and methodological quality

Study design and quality can affect which study results are considered appropriate to combine in a systematic review. The QUADAS-2 tool is the internationally recognised tool to assess the methodological quality (both risk of bias and applicability) of DTA studies [11]. QUADAS-2 suggests study quality information can be integrated directly into the review analysis, by including a meta-analysis of studies providing the strongest evidence (lowest risk of bias, highest applicability). QUADAS-C, the risk of bias tool for comparative diagnostic accuracy studies [12], can be used similarly.

For the study design domain, Table 2 identifies three types of variation between studies: (1) unit of analysis (2); risk of bias ratings from QUADAS-2/QUADAS-C ratings or individual sources of bias; and (3) applicability ratings from QUADAS-2.

Study results in a systematic review can refer to participants, samples, lesions, organs, images or hospital visits. The unit of analysis identifies who/what the results refer to, for example whether the test accuracy results are reported using the number of participants or, if a participant can have more than one image, the number of images. Sometimes a systematic review will include studies with results using more than one unit. For example, an imaging test to identify polyps in the colon could report the accuracy to identify a person with polyps, or the accuracy to identify a polyp [13].

Accuracy per participant is important if the aim of the test is to identify the right patients for further tests and interventions. Accuracy per polyp is important for tests such as colonoscopy, which aim to identify and at the same time treat polyps, to understand if all relevant polyps within a patient would be treated. In a review estimating the accuracy of CT colonoscopy, per polyp analyses were based on polyp size (large, medium, all size), pre-specified from clinical guidelines according to treatment recommendations, so data were extracted and reported by polyp size [13].

In the two example reviews of typhoid and ovarian cancer, it is only clinically relevant to consider test accuracy based on participants as blood tests can only provide results across all potential disease sites within a patient.

In both the typhoid and ovarian cancer reviews, the QUADAS-2 signalling question about study design was used to understand how a key potential source of bias might affect results with planned heterogeneity analysis and presentation based on study design being case-control or not case-control.

## Step 3: Simplifying a review

The aims of step 3 are to simplify a review, by combining complexities within an analysis where possible without compromising clinical relevance, and to enable more efficient planning of the review. Decisions and the reasons underlying them are recorded in the column ‘step 3’ of Table 2. Identifying groups of participants, index tests or target conditions where it is essential to have separate analysis requires good communication between members of the review team with clinical and methodological expertise. 

At the same time, it is important to minimise the number of separate main analyses, or the review can quickly become a descriptive analysis of individual studies. Investigations of heterogeneity, sensitivity analyses and graphical presentation of data are other useful ways of exploring and understanding the effects of different aspects of the complexity of a review. Some elements of complexity may not be considered clinically relevant to a particular review so that it is not necessary to present data separately in graphs or analyses, while other sources of clinical variability may be important to retain. 

**Fig. 1:**
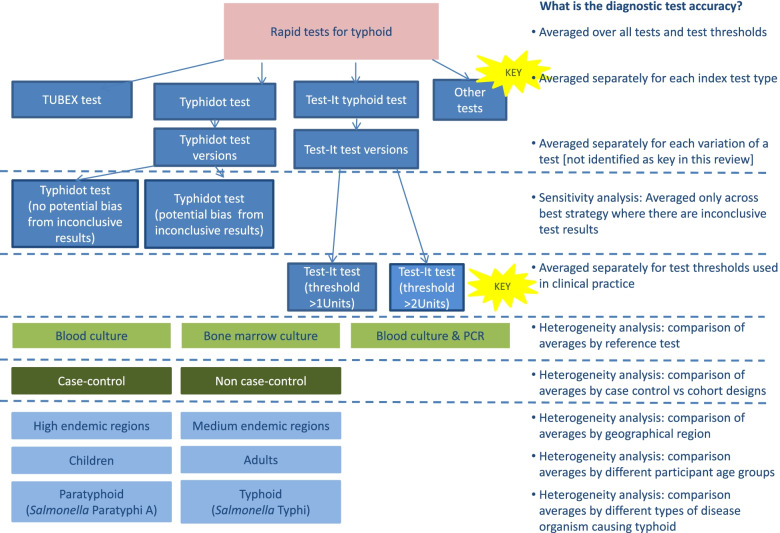
Flowchart of planned analyses: Typhoid review. Different components of variation leading to complexity in review of typhoid rapid tests. Coloured boxes indicate the TOMAS-R domain where complexity identified: pink boxes review topic; light blue boxes participant domain; dark blue boxes index test domain; light green boxes target condition domain; dark green boxes study design and quality domain. Dotted lines separate complexity and allow alignment to the diagnostic accuracy that the analysis would address. Each bullet point follows the question “What is the diagnostic accuracy...” so for example if all rapid test results are combined the first bullet point is used so the analysis will answer the question “What is the diagnostic accuracy *averaged over all tests and test thresholds*?” Yellow stars indicate key complexities identified as requiring separate analyses for the review to have clinical relevance

**Fig. 2:**
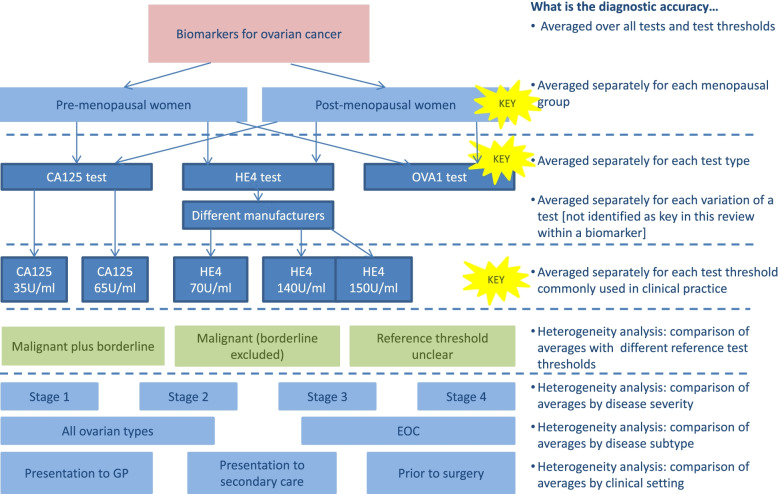
Flowchart of planned analyses: Ovarian cancer serum biomarker review. Different components of variation leading to complexity in review of serum biomarkers in ovarian cancer. Coloured boxes indicate the TOMAS-R domain where complexity identified: pink boxes review topic; light blue boxes participant domain; dark blue boxes index test domain; light green boxes target condition domain. Dotted lines separate complexity and allow alignment to the diagnostic accuracy that the analysis would address. Each bullet point follows the question “What is the diagnostic accuracy...” so for example if test results are separated by the menopausal status of the women, the second bullet point is used so the analysis will answer the question “What is the diagnostic accuracy *averaged separately for each menopausal group*?” Yellow stars indicate key complexities identified as requiring separate analyses for the review to have clinical relevance

We recommend a flowchart of studies is used to identify how different review questions are answered depending on how complexity is combined or separated in subgroups. As studies are subdivided into separate subgroups for meta-analysis, the question answered by the meta-analysis is different. We present flowcharts for our two example reviews (Figs. 1 and 2).

For the typhoid review, the clinical members of the review team deemed it important to investigate diagnostic test accuracy separately for each of the main commercial tests, simplifying the review by combining different versions of the same test. For the Test-It typhoid test where there were two thresholds, separate analysis was required at each test threshold so that no simplification of thresholds was possible. Other sources of variation can be presented graphically, or where there were sufficient studies as heterogeneity or sensitivity analyses.

## Step 4: Planning data extraction

The aim of step 4 is to identify if any complexity in the data affects data extraction. Using a separate column ensures that discussion between methodologists and clinical experts consider and record all these decisions during the review planning.

We recommend researchers design and pilot a standardised data extraction sheet with explanations of what should be extracted and how missing information and inconclusive test results will be handled.

Data extraction can be speeded up and made more consistent if all team members understand and use the same methods so only clinically important categories are extracted separately. Common data extraction issues arise when studies report test results at multiple time points or at multiple test thresholds. If a study reports 20 different results, it is possible that not all thresholds or time points are relevant to the review question.

In the ovarian cancer review, studies used different reference standard thresholds. Data extraction was completed using a priority order to reflect the most important results for achieving the review aims. We also speeded up our data extraction by deciding to extract results only for commonly used and clinically relevant index test thresholds. Data were not extracted where the index test threshold was not reported as these results cannot inform clinical practice. In the typhoid review, data extraction was simplified based on pre-specification of the reference standard according to standard definitions in the typhoid literature, as grade 1 or grade 2 and included pre-specified rules on how multiple tests within the reference standard would be considered.

## Step 5: Planning presentation and analysis of data

Once decisions have been made on simplifying a review (step 3, Fig. 1 and Table 2) and which data to extract (step 4, Table 2), planning the analysis follows from these decisions, and the practical realities of the number of studies in analysis groups and subgroups.

The TOMAS-R template includes a column to record the planned presentation and analyses for each issue raised in the review using the column ‘step 5’ of the TOMAS-R template (Table 2). As in other statistical analyses, primary and secondary outcomes need to be identified. Some of these will include analysis if there are sufficient data, but some outcomes may focus on graphical display of data. The Cochrane DTA Handbook includes details of methods for meta-analysis and how to present the results (e.g. displaying summary points (with confidence and prediction regions on SROC plots) and investigation of heterogeneity. Example software code is provided for different statistical software packages [5, 14].

### Choosing presentation and analysis

There are three main types of analysis in DTA reviews: (1) meta-analysis of a single index test; (2) meta-analysis to compare the accuracy of two or more index tests; and (3) investigation of heterogeneity. The first two types of analysis are usually the primary analyses. Presentation of data alongside the analysis facilitates clarity and transparency and this may be done graphically or in a tabular format as appropriate. Network meta-analysis methods are available but currently not widely used [15, 16].

Where index tests are compared, the strongest evidence is based on a direct comparison within the same study, either where both tests are completed on the same participants (paired study data), or where participants with the two tests are as similar as possible, i.e. participant is randomised to each test [17, 18]. It is important that an analysis plan states whether comparisons of tests will be based on direct comparisons using comparative accuracy studies or on all available data, including data from studies that assessed only one of the index tests (indirect comparisons).

Presentation of data in a review typically includes graphical display of results using SROC and forest plots. Both graphs allow both sensitivity and specificity results to be displayed, with forest plots providing a clearer display of 95% confidence intervals (CIs) when there are a large number of studies. Although 95% CIs can be displayed on SROC plots, once there are several overlapping studies, the plot becomes overcrowded and unclear. Paired study results can be displayed in an SROC plot with a line linking results from the same study.

### Guidance on investigation of heterogeneity and sensitivity analysis

Investigation of heterogeneity is used to determine how test accuracy varies with clinical and methodological characteristics, whereas sensitivity analysis is used to understand how robust the main study results are to decisions made during the review process.

To understand whether study characteristics affect study results, investigations of heterogeneity can be performed. Graphical displays of subgroups in SROC or forest plots allows visual inspection for potential heterogeneity. This is particularly important when it is not possible to statistically investigate heterogeneity due to the inclusion of a small number of studies in the primary meta-analyses. In a heterogeneity analysis involving a categorical variable, the dataset will consist of non-overlapping subgroups which may be statistically compared in a meta-analysis (meta-regression) or an analysis is performed for each subgroup separately (subgroup analyses). This contrasts to sensitivity analyses where meta-analysis is repeated using a subset of studies, in order to assess the robustness of the findings to assumptions made during the review process. Both heterogeneity and sensitivity analyses should be pre-planned in the review protocol.

In the typhoid review, investigation of heterogeneity analysis was planned to examine the role of nine study characteristics including disease endemicity of typhoid, geographical region and index test format. However, there were insufficient studies to examine any of these in a statistical analysis, although an SROC graph was used to display studies according to type of reference standard and study design.

By contrast, the typhoid review included a sensitivity analysis restricted to studies of the rapid test typhidot where there was a low bias expected from inconclusive test results, caused by conflicting results from IgG and IgM antibodies. The ovarian cancer review was only able to complete planned heterogeneity analyses comparing studies including borderline results as part of the reference standard, as opposed to studies either unclear or specifically excluding borderline test results.

### Guidance on index test thresholds in meta-analysis

A common mistake in DTA reviews that compromises the clinical relevance is to combine test results across very different thresholds for defining a positive test result by using methods that allow only one threshold per study for the estimation of an average sensitivity and specificity. Results combined across very different thresholds in this way do not give a result that can be interpreted at any clinically relevant threshold, but correspond to an average result reflecting how often different thresholds are reported. For example, in the typhoid review, it is important not to combine results from the two thresholds of the Test-It test.

Therefore, the choice of a meta-analysis method depends on the type of data available and the focus of interest. If studies report a common threshold, estimating an average sensitivity and specificity (summary point) at that threshold is appropriate. However, if studies report different thresholds, estimating a SROC curve across different thresholds by including one threshold per study is more appropriate. If some or all of the studies report more than one threshold, more complex methods that produce SROC curves across the thresholds as well as estimates of average sensitivity and specificity at specific thresholds can be used to make the most of the available data as well as to identify a relevant threshold that meets a desired level of test performance [9, 10]. The DTA Cochrane handbook provides guidance on data extraction [19] and meta-analysis with multiple test thresholds [14].

## Including TOMAS-R in systematic review protocols

TOMAS-R is suitable as a tool to guide planning in a review and to maintain communication within a team, but also to provide a clear summary table of review planning for inclusion in a systematic review protocol. Clearly, it is not possible to plan for all eventualities in a review protocol, and TOMAS-R could also be used to report changes between the protocol and final review.

## Concluding remarks

DTA systematic reviews require careful planning to enable them to address clinical objectives in an informative way. Careful planning is facilitated by a structured approach, particularly in DTA reviews where there is often considerable complexity due to variations between studies.

TOMAS-R is a template to allow structured planning with prompts to identify sources of complexity identified as common in DTA systematic reviews. In this article we have described how this template can be used during protocol development for planning DTA reviews. We anticipate this template will enhance the quality and consistency of protocols by providing a structured approach, similar to tools and checklists already in use, such as reporting guidelines and risk of bias tools. An earlier version of this template has been adapted for prognostic reviews [20], using terminology used in prognostic SRs. A blank template table is provided in supplementary materials (Table S1).

The template can also be used for reporting what was done in a review and changes between the protocol and the review. In addition, we have also found the template is useful for peer review of DTA and prognostic reviews, either at the protocol or full review stage.

As with other checklists and tools in medical research, TOMAS-R and its guidance will require updating as methods for diagnostic accuracy studies develop and further validation is undertaken. We recommend downloading the latest version of TOMAS-R and accompanying guidance, including detailed examples, from the OSF open repository site (https://osf.io/).

## Supplementary Information

**Additional file 1: Table S1.** TOMAS-R template to identify clinically important variation and multiplicity

## Data Availability

Not applicable

## Abbreviations

CA125: Cancer antigen 125
CIs: Confidence intervals
DNA: Deoxyribonucleic acid
DTA: Diagnostic test accuracy
ELISA: Enzyme-linked immunosorbent assay
EOC: Epithelial ovarian cancer
GP: General practitioner (primary care clinician)
HE4: Human epididymis protein 4
IgG: Immunoglobulin G
IgM: Immunoglobulin M
NIHR: National Institute for Health Research
OVA1: Ovarian cancer test OVA1
PCR: Polymerase chain reaction
QUADAS-2: Quality assessment of diagnostic accuracy studies
QUADAS-C: Quality assessment of diagnostic accuracy studies comparison
RDTs: Rapid diagnostic tests
ROC: Receiver operating characteristic
SROC: Summary receiver operating characteristic
ToMAS-R: Template of Multiplicity and Analysis in Systematic Reviews
TOC: Table of study characteristics
