# Supplementary material for: TOMAS-R: A template to identify and plan analysis for clinically important variation and multiplicity in diagnostic test accuracy systematic reviews
Source: Diagn Progn Res. 2022 Sep 22;6:18. doi: 10.1186/s41512-022-00131-z (PMC9494799; doi:10.1186/s41512-022-00131-z)
Supplement: Supplementary file 1 — Additional file 1: Table S1. TOMAS-R template to identify clinically important variation and multiplicity [file 41512_2022_131_MOESM1_ESM.doc]

# Supplementary material

# Table S1: Example TOMAS-R template to identify clinically important variation and multiplicity for systematic reviews

| **SUMMARY OF REVIEW (STEP 1)**  **TITLE:**  **PRIMARY OBJECTIVE:**  **SECONDARY OBJECTIVES:** |
| --- |
| **PARTICIPANTS:**  **INDEX TEST(S):**  **ROLE OF TEST FOR PATIENT** (Delete as appropriate DIAGNOSIS, MONITORING, STAGING):  **ROLE OF TEST IN PLANNED CLINICAL PATHWAY:** (Delete as appropriate TRIAGE, ADD ON, REPLACEMENT):  **TARGET CONDITION:**  **REFERENCE STANDARDS:**  **STUDY DESIGNS:** |

| **DOMAIN 1: PARTICIPANTS** | | | | |
| --- | --- | --- | --- | --- |
| **Potential sources of clinically important complexity** | **STEP 2**  **List categories identified from review scoping** | **STEP 3**  **Report which categories will be separate or combined. Give Reason** | **STEP 4**  **Data extraction: Report if any categories will be preferentially extracted.** | **STEP 5**  **Presentation and meta-analysis: Report how categories will be treated** |
| **1.1: Clinical pathway/ Prior tests/Different comorbidities/Geographical regions**  Are there important differences between participants that could affect test accuracy?  Examples   - Different clinical pathways or healthcare settings (primary care, secondary, tertiary care) - Different prior tests (referral based on different prior tests) - Differences in other conditions likely to be present at same time - Different geographical settings | **Clinical pathway/ Prior tests** |  |  |  |
|  | **Different comorbidities** |  |  |  |
|  | **Geographical location** |  |  |  |
| **1.2: Disease type or severity**  Are there groupings within participants by disease type or severity that could affect test accuracy?  Example  • Different severity of disease: patients with mild disease vs with severe disease  • Different disease state e.g. active vs past disease (inactive)   - Different types of diseased e.g. pigmented vs non-pigmented lesions in skin cancer |  |  |  |  |
| **1.3: Participant demographics**  Are there any important groupings by participant age, gender, ethnicity?  Example separate groups by  • Different ages such as children and adults  • Different demographics such as gender, ethnicity, genetic groups |  |  |  |  |

| **DOMAIN 2: INDEX TEST(S)**  **CRITERIA USED TO FOCUS REVIEW TO MOST CLINICALLY RELEVANT TEST(S)**: | | | | | | | | |
| --- | --- | --- | --- | --- | --- | --- | --- | --- |
| **Reason for potential groupings or categories.** | | **STEP 2**  **List categories identified from review scoping** | **STEP 3**  **Report which categories will be separate or combined. Give Reason** | | **STEP 4**  **Data extraction: Report if any categories will be preferentially extracted.** | | **STEP 5**  **Presentation and meta-analysis: Report how categories will be treated** | |
| **2.1 Type of underlying index test**  Is more than one underlying type of index test included that could affect test accuracy?  Examples   - Different indications of disease presence e.g. DNA of infectious agent, antibodies against infectious agent. - Different formats of test e.g. ELISA, PCR, dipstick - Different equipment needed that affect test e.g. laboratory test using specialist equipment, point of care test. |  | | |  | |  | |  |
| **2.2 Index test methods within an index test grouping**  Is there more than one method or manufacturer for a test that could affect test accuracy?  Also consider if the test might be done by people with different level of experience or using different approaches to interpretation.  **Examples**   - Different test versions of tests - Different participant samples used to detect disease e.g. blood sample, urine sample - Differences in staff e.g. trained laboratory staff vs nurse point of care test - Different treatment of inconclusive test results - Different approach to assist test interpretation, e.g. algorithms or checklists | **Different test versions** | | |  | |  | |  |
|  | **Different samples** | | |  | |  | |  |
|  | **Different treatment of inconclusive results** | | |  | |  | |  |
| **2.3 Threshold(s) for positive index test result**  Are different thresholds used to define a positive result that could affect test accuracy?  Has a clinically relevant index test threshold been identified for this review?  Examples   - Different test thresholds used to define a positive test result for semi-quantitative or continuous test results |  | | |  | |  | |  |

| **DOMAIN 3: TARGET CONDITION** | | | | | | | | |
| --- | --- | --- | --- | --- | --- | --- | --- | --- |
| **Reason for potential groupings or categories.** | | **STEP 2**  **List categories identified from review scoping** | **STEP 3**  **Report which categories will be separate or combined. Give Reason** | | **STEP 4**  **Data extraction: Report if any categories will be preferentially extracted.** | | **STEP 5**  **Presentation and meta-analysis: Report how categories will be treated** | |
| **3.1 Types of target condition**  Are there different target conditions included that could affect test accuracy?  **Examples**   - Different causes of disease (e.g. different organisms causing typhoid infection, different causes of trauma injury) - Different types or severity of disease that are treated differently e.g. malignant and borderline disease in ovarian cancer disease diagnosis, any melanoma or melanoma with high potential to progress to malignancy |  | | |  | |  | |  |
| **3.2 Reference standards**  Are different methods used to verify disease presence or absence that could affect test accuracy?  **Examples**  • Different methods to detect typhoid infection measured by detection of viral DNA or by bacterial culture |  | | |  | |  | |  |

| **3.3 Thresholds for reference standard**  Are different criteria or thresholds used to define presence of disease that could affect test accuracy?  Example  • Different definitions of fasting blood glucose to define diabetes |  |  |  |  |
| --- | --- | --- | --- | --- |
| **3.4 Time of reference standard determination**  Are there differences in when reference standard is completed that that could affect test accuracy?  Examples   - Different time points reference standard assessed - Different maximum or minimum time intervals between reference standard and index test |  |  |  |  |

| **DOMAIN 4: STUDY DESIGN AND QUALITY** | | | | | | | |
| --- | --- | --- | --- | --- | --- | --- | --- |
| **Reason for potential groupings or categories.** | **STEP 2**  **List categories identified from review scoping** | **STEP 3**  **Report which categories will be separate or combined. Give Reason** | | **STEP 4**  **Data extraction: Report if any categories will be preferentially extracted.** | | **STEP 5**  **Presentation and meta-analysis: Report how categories will be treated** | |
| **4.1: Unit of analysis:**  Are there differences in whether test results are a single test or more than one test result per participant (unit of analysis)?  **Examples**   - Test results could refer to individual participants, lesions, organ, clinic visits or imaging scans |  | |  | |  | |  |
| **4.2: Risk of bias: QUADAS-2/QUADAS-C item or domain**  Based on risk of bias assessed using QUADAS-2/QUADAS-C, are there important differences between that could affect test accuracy?  **Examples**   - Single signalling question e.g. specific design criteria (case control vs better design using cohort or nested case control) - Differences in QUADAS-2/QUADAS-C overall domain assessment of bias e.g. participant domain |  | |  | |  | |  |

| **4.3: Applicability: QUADAS-2 item or domain**  Based on applicability of study results assessed using QUADAS-2, are there important differences between studies that could affect test accuracy?  Applicability of participants could depend on several factors and might be best summarised by analysis grouped by applicability of the participant recruitment assessed in QUADAS-2  **Example** Differences in QUADAS-2 domains for applicability of:   - Participants - Index tests - Reference standard |  |  |  |  |
| --- | --- | --- | --- | --- |

**Example footnotes that can be used to add analysis details**

^1^Meta-analysis will only be done if (i) there are four or more studies where results are given in the same format (e.g. 2x2 table for diagnosis) (ii) study results are sufficiently homogeneous visualised in forest plots or ROC space for a meaningful representation by a single summary statistic.

^2^Priority order of data extraction means that not all data will be extracted from published articles.

^3^To avoid over representing results from a study in meta-analysis results, we will include only one set of results per index test from each study.
